# Evolutionary relevance of single nucleotide variants within the forebrain exclusive human accelerated enhancer regions

**DOI:** 10.1186/s12860-023-00474-5

**Published:** 2023-03-29

**Authors:** Hizran Khatoon, Rabail Zehra Raza, Shoaib Saleem, Fatima Batool, Saba Arshad, Muhammad Abrar, Shahid Ali, Irfan Hussain, Neil H. Shubin, Amir Ali Abbasi

**Affiliations:** 1 National Center for Bioinformatics, Program of Comparative and Evolutionary Genomics, Faculty of Biological Sciences, Quaid-I-Azam University, Islamabad, 45320 Pakistan; 2 Department of Biological Sciences, National University of Medical Sciences, Rawalpindi, 46000 Pakistan; 3 Department of Organismal Biology and Anatomy, The University of Chicago, Chicago, IL USA

**Keywords:** Evolution, Enhancers, Human accelerated regions, Forebrain, Archaic hominins, TFBS, SNVs, SOX2, HMG box, EMSA

## Abstract

**Background:**

Human accelerated regions (HARs) are short conserved genomic sequences that have acquired significantly more nucleotide substitutions than expected in the human lineage after divergence from chimpanzees. The fast evolution of HARs may reflect their roles in the origin of human-specific traits. A recent study has reported positively-selected single nucleotide variants (SNVs) within brain-exclusive human accelerated enhancers (BE-HAEs) hs1210 (forebrain), hs563 (hindbrain) and hs304 (midbrain/forebrain). By including data from archaic hominins, these SNVs were shown to be Homo sapiens-specific, residing within transcriptional factors binding sites (TFBSs) for SOX2 (hs1210), RUNX1/3 (hs563), and FOS/JUND (hs304). Although these findings suggest that the predicted modifications in TFBSs may have some role in present-day brain structure, work is required to verify the extent to which these changes translate into functional variation.

**Results:**

To start to fill this gap, we investigate the SOX2 SNV, with both forebrain expression and strong signal of positive selection in humans. We demonstrate that the HMG box of SOX2 binds in vitro with Homo sapiens-specific derived A-allele and ancestral T-allele carrying DNA sites in BE-HAE hs1210. Molecular docking and simulation analysis indicated highly favourable binding of HMG box with derived A-allele containing DNA site when compared to site carrying ancestral T-allele.

**Conclusion:**

These results suggest that adoptive changes in TF affinity within BE-HAE hs1210 and other HAR enhancers in the evolutionary history of Homo sapiens might.

have brought about changes in gene expression patterns and have functional consequences on forebrain formation and evolution.

**Methods:**

The present study employ electrophoretic mobility shift assays (EMSA) and molecular docking and molecular dynamics simulations approaches.

**Supplementary Information:**

The online version contains supplementary material available at 10.1186/s12860-023-00474-5.

## Background

The regulation of gene expression during embryogenesis is a cornerstone for the formation and evolution of complex metazoan life [1]. Much of the genetic information concerning gene regulation is encoded by cis-regulatory DNA enhancers [2]. Enhancers are generally defined as short non-coding DNA segments, typically 100–1,000 base-pair (bp) in length that govern target gene transcription over short to long genomic distances [2]. Expression of a typical eukaryotic gene is likely to be regulated by multiple different enhancers that can be reside in the 5ˊ and 3ˊ genomic regions, as well as within intronic intervals of other genes [3]. Such modular organization of gene regulatory networks allows each enhancer to govern a subset of the total gene expression pattern for a particular gene. Furthermore, each enhancer usually mediates expression within a specific tissue or cell type or during specific developmental stage or domain [4]. Given the importance of enhancers during embryonic development, the genetic alterations in these non-coding sequences could result in phenotypic alterations [5].

In addition to their central roles in development and disease, enhancers are fertile targets for evolutionary modifications [6]. Key understanding of enhancer functions during evolutionary divergence among animal forms have come from *Drosophila* genetics [7]. Fueled by the increasing availability of genome sequences, technological progress in the fields of genomics, genome editing and comparative genomics, recent years have seen a renewed interest in elucidating roles of non-coding enhancers in evolution of diverse species, including humans [8]. We now know that the vast majority of all genomic differences that happened since human and chimpanzees diverged ~ 6 million years ago (Mya) are in non-coding regions [9]. The challenge in the post-genomic era has been to determine human-specific non-coding regulatory variants that are responsible for evolution of Homo sapiens biology [9]. The large size of the non-coding genome makes identifying functionally-relevant genetic variants challenging. To overcome this hurdle, a number of comparative genomics based strategies have been devised to identify non-coding DNA sequences with dramatically increased substitution rates in the human lineage when compared to our closest living relatives, chimpanzees and bonobos or that arose after divergence from archaic hominids [10, 11]. These loci are termed as Human Accelerated Regions (HARs), are short, ~ 260 bp in length, ~ 97% of them are non-coding [12]. On the basis of sequence features and functional data, a recent investigation predicted that at least one third of non-coding HARs function as enhancers active in many different embryonic tissues [13]. The fact that HARs are significantly different between humans and their closely related species with majority of them being regulatory enhancers, suggests their potential association to some human-specific traits [14]. Indeed, several studies have associated HARs in regulatory regions with diverse neurodevelopmental phenotypes such as Autism Spectrum Disorders (ASDs), microcephaly, seizures and impairments of language and speech development [15, 16]. These data highlight the significance of HARs in human-specific neural evolution and diversification.

**Fig. 1:**
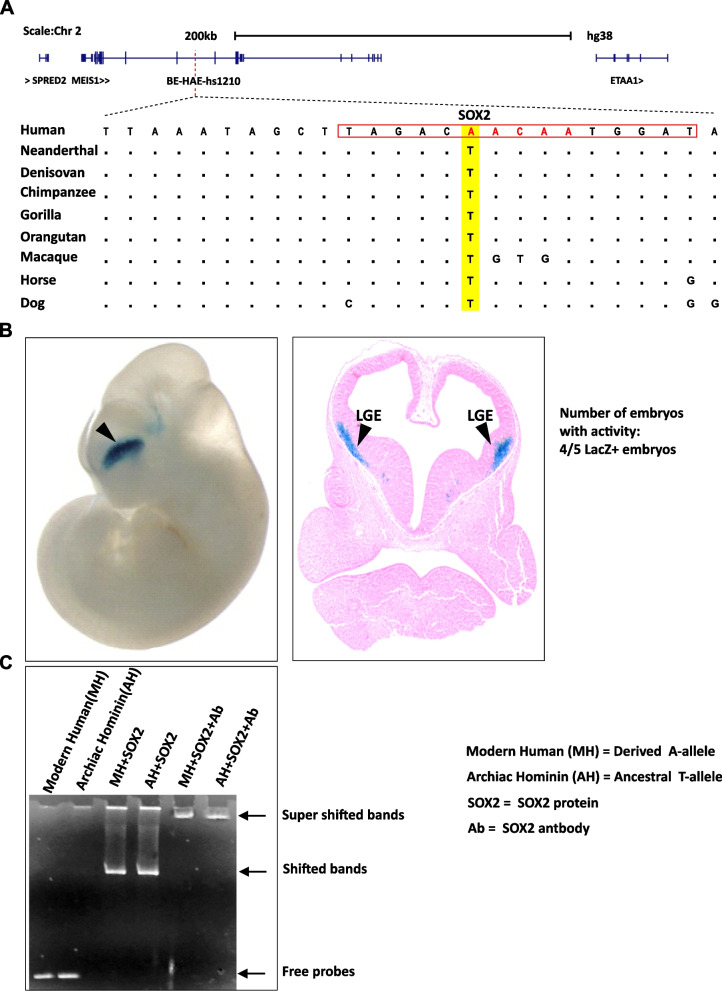
Comparative sequence and functional analysis of human accelerated enhancer hs1210. **A** The BE-HAE-hs1210 is located within the intron of MEIS1 gene on chromosome 2. The alignment illustrates the Homo sapiens-specific substitution in the non-coding HAR. The red line indicates the position of the BE-HAE-hs1210 within the intron of MEIS1 gene. The red rectangle within the alignment highlights the SOX2 binding motif, and the red highlighted nucleotides are the core motif of SOX2. Conserved nucleotides are depicted as dots. **B** BE-HAE-hs1210 induced LacZ expression in transgenic mouse embryo at day E11.5. Whole mount embryo at E11.5 depict LacZ expression in the mouse forebrain (black arrow-head). Cross section of mouse embryonic forebrain revealed the BE-HAE-hs1210 enhancer activity in the sub-pallial region (LGE, lateral ganglionic eminence) (black arrow-head). Whole mount and cross section data of mouse is obtained from [19]. **C** Electrophoretic mobility shift assay (EMSA) or Gel shift assay shows shift in the mobility of SOX2 protein-DNA complexes as compared to the free probes (Homo sapien and archaic hominin). Binding of SOX2 protein hinders the mobility of DNA probe (shifted bands) and addition of antibody to SOX2 bound DNA probe further reduced the mobility of complex in the gel (super shifted bands). Full size uncropped version of Fig. 1C is provided as Additional file 3

Using human-chimpanzee alignments, it has been shown that human-specific nucleotides alter the transcriptional regulatory potential of brain-specific regulatory HARs through gain and loss of important transcription factors binding sites (TFBSs) [16, 17]. Therefore, the comparative analysis of HARs for gain and loss of TF binding motifs can be used to identify candidate HARs and their specific nucleotides regulating human-specific neural developmental processes such as higher cognitive functions, synaptic complexity and the overall brain size. In one such attempt computational and statistical approaches were employed to shortlist among the empirically confirmed brain exclusive enhancers the ones with an accelerated rate of sequence divergence in the human lineage [18]. These were termed as brain exclusive human accelerated enhancers (BE-HAEs). In this set of human accelerated enhancers, derived from comparisons with archaic hominins, three distinct Homo sapiens unique SNVs were identified that potentially altered the putative binding sites of transcription factors SOX2, RUNX1/3, and FOS/JUND within BE-HAEs hs1210 (forebrain), hs563 (hindbrain) and hs304 (midbrain/forebrain) respectively [18]. Furthermore, the haplotype-based statistical analysis laid out evidence of positive selection in extant human population on the three SNVs with strongest results for haplotypes containing Homo sapiens-specific A-allele within the SOX2 binding site. Therefore, a Homo sapiens-specific derived binding site (TAGACA^*^ACAATGGAT) was evolved within forebrain exclusive BE-HAEs hs1210 as compared to the ancestrally conserved site (TAGACT^*^ACAATGGAT) (Fig. 1A).

To enhance understanding of the evolutionary changes controlling gene expression differences between Homo sapiens and archaic hominins, we have investigated the structural impact of positively selected TF binding nucleotide residues within regulatory HARs. We used the SOX2 binding site within forebrain exclusive HAE hs1210 which differ from the orthologous site in archaic hominins (Neanderthal/Denisovan) by a single nucleotide residue (Fig. 1A and B). This region has the strongest signal of positive selection in Homo sapiens [18]. We characterized the allelic variants of SOX2 binding site within HAEs hs1210 by using the electrophoretic mobility shift assay–based measurement of TF binding and by using molecular docking and simulation approaches. We showed that Homo sapiens-specific nucleotide variants enhanced the ability of the TF SOX2 to bind to its target DNA site, indicating it might have modified its ability to drive transcription in forebrain tissues. Our findings add to the growing body of evidence suggesting that single nucleotide changes within non-coding regulatory HARs may be responsible for Homo sapiens brain phenotype and neural developmental processes.

## Results

### In vitro binding analysis of SOX2 to target DNA containing ancestral and derived alleles

Electrophoretic mobility shift assays (EMSA) were performed using the purified SOX2 protein and two de novo pairs of complementary oligonucleotides, carrying the ancestral T-allele (Neanderthal/Denisovan) and derived A-allele (Homo sapiens) (Additional files 1 and 2. It appeared that both ancestral and derived alleles containing oligonucleotides were capable of binding to the SOX2 protein and thus migrates through a polyacrylamide gel more slowly than the corresponding free unbound DNA (Fig. 1C and Additional file 3). Addition of antibodies directed against SOX2 caused further retardation (EMSA super shift protocol) within the gel and thus confirmed that the bound protein in these complexes is SOX2 (Fig. 1C). Therefore, based on EMSA protocol it can be suggested that SOX2 is capable of binding with the derived (TAGACA*ACAATGGAT) as well as the ancestral (TAGACT*ACAATGGAT) versions of its target DNA sites (Fig. 1A).

### Molecular docking characterization of the protein-DNA complex

**Fig. 2:**
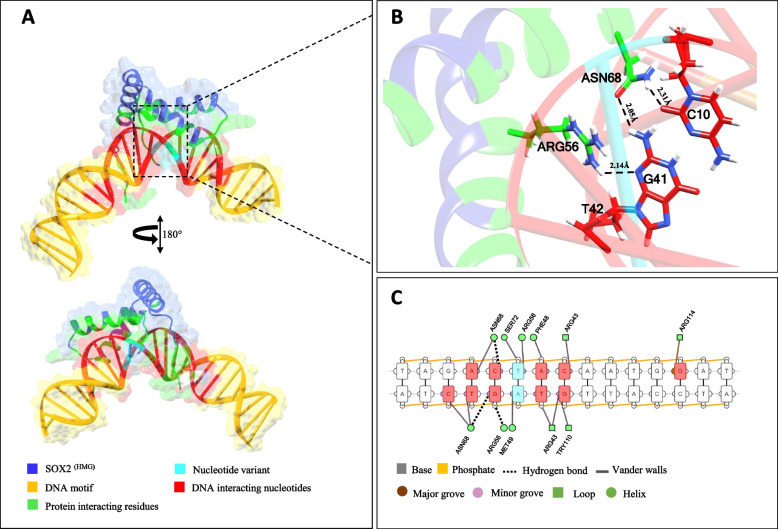
Structural analysis of Ancestral ^T−allele^ DNA- SOX2 ^(HMG)^ complex. **A** Topological model for SOX2 ^(HMG)^ binding to ancestral ^T−allele^ DNA shown as semi-transparent surface and ribbons. **B** The zoomed image illustrates the interface between the residues of SOX2 ^(HMG)^ and corresponding nucleotides. Hydrogen bonds are shown by black dotted lines with calculated distances in angstrom (Å). **C** Schematic diagram depicts interactions between Ancestral ^T−allele^ DNA- SOX2 ^(HMG)^ complex. Nucleotides (red) are interacting with amino acid residues (green) of SOX2 ^(HMG)^ through hydrogen bonds (dotted lines) and Vander walls (solid lines). The DNA strands are displayed as orange. Cyan color shows the position of nucleotide variant in SOX2 binding site. Images were obtained from DNAproDB

**Fig. 3:**
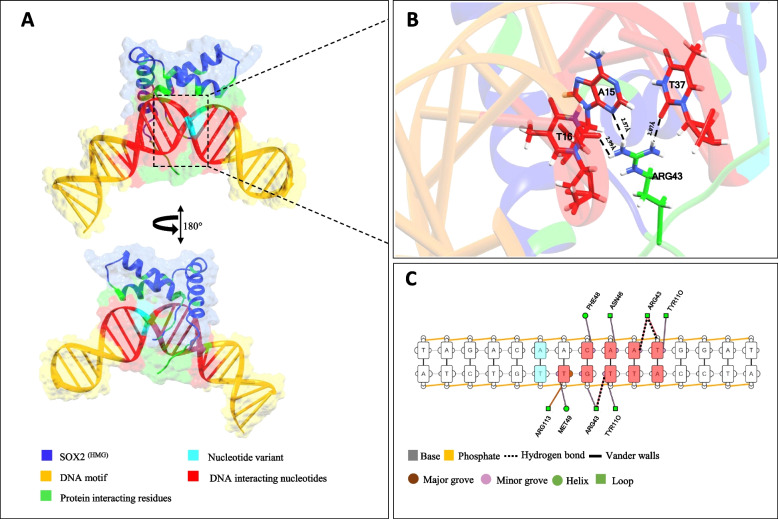
Structural analysis of Derived ^A−allele^ DNA- SOX2 ^(HMG)^ complex. **A** Topological model for SOX2 ^(HMG)^ binding to derived ^A−allele^ DNA shown as semi-transparent surface and ribbons. **B** The zoomed image illustrates the interface between the residues of SOX2 ^(HMG)^ and corresponding nucleotides. Hydrogen bonds are shown by black dotted lines with calculated distances in Å. **C** Schematic diagram depicts interactions between derived^A−allele^ DNA- SOX2 ^(HMG)^ complex. Nucleotides (red) are interacting with amino acid residue (green) through hydrogen bonds (dotted lines) and Vander walls (solid lines). The DNA strands are displayed as orange. Cyan color shows the nucleotide variant in the Derived DNA binding site. Images were obtained from DNAproDB

Comparative sequence analysis showed that the DNA binding HMG box of SOX2 (SOX2 ^(HMG)^) is highly conserved among Homo sapiens, archaic humans, non-human primates and other mammals (Additional file 4)**.** Molecular docking was performed to obtain an atomic level understanding of conformational alterations in protein-DNA complex upon the binding of HMG box to ancestral (Neanderthal/Denisovans) and derived (Homo sapiens-specific) target sites (Fig. 1A). The molecular docking results corroborates well with in vitro data and revealed the binding of SOX2 ^(HMG)^ with both target sites carrying ancestral T-allele or derived A-allele (Figs. 2A and 3A). However, careful analysis has revealed the notable conformational and energetic differences between the two complexes (Figs. 2B and 3B, Table 1). For instance, in corroboration with previously reported experimental data our molecular docking results indicate that the HMG box of SOX2 protein (SOX2 ^(HMG)^) grips directly the minor groove of the double helix DNA structure for both ancestral T-allele and derived A-allele containing target sites (Figs. 2A and 3A, Table 2) [20, 21]. However, nucleotides with which the SOX2 ^(HMG)^ interacts directly are slightly different for ancestral T-allele containing target DNA site (5′-GACT^*^AC-3′) and derived A-allele (Homo sapiens-specific) containing target DNA site (5′-ACAAT-3′) (Figs. 2C and 3C). Intriguingly, the ancestral T-allele was involved in direct interaction with SOX2 ^(HMG)^, however its mutant version in Homo sapiens, i.e. derived A-allele did not interact directly with SOX2 ^(HMG)^ (Figs. 2C and 3C). Furthermore, noticeably different types of amino acid residues and secondary structural elements (SSEs) of HMG box were involved in interactions with ancestral and derived target sites (Table 2). It appears that Homo sapiens-specific nucleotide substitution has changed the binding conformation and location of target DNA site for HMG box. Energetic profile evaluation revealed higher affinity of SOX2 ^(HMG)^ for the derived A-allele carrying target DNA site (-281.6 ± 4.2 kcal/mol), whereas relatively lower affinity was noticed for the ancestral T-allele carrying target DNA site (-270.3 ± 4.0 kcal/mol) (Table 1).

**Table 1:** Molecular docking based energetic profile evaluation through HADDOCK

Docked complex	HADDOCK score	Z-score	Van der Waals energy	Electrostatic energy	Desolvation energy	Restraints violation energy	Buried surface area in Å^2^
Derived A-allele DNA-SOX2	-281.6 ± 4.2	0	-97.7 ± 2.5	-1061.0 ± 36.3	28.0 ± 3.2	2.3 ± 0.23	2622.1 ± 39.6
Ancestral T-allele DNA -SOX2	-270.3 ± 4.2	0	-110.6 ± 2.7	-892.6 ± 21.5	18.7 ± 3.7	1.7 ± 0.31	2868.9 ± 27.4

All the energies are calculated as kcal/mol. The Haddock score is lower for derived^A−allele^ DNA- SOX2complex (-281.6 ± 4.2 kcal/mol) as compared to ancestral ^T−allele^ DNA-SOX2 complex (-270.3 ± 4.0 kcal/mol)

**Table 2:** DNA binding residues of SOX2-HMG box reported previously (experimentally determined) and in the present study

	**Remenyi et.al. 2003** **DNA-HMG box**		**Present study** **A-allele DNA- HMG box**		**Present study** **T-allele DNA- HMG box**	
Sr. No	Interacting residues	SSEs	Interacting residues	SSEs	Interacting residues	SSEs
1	ARG43	L1	ARG43	L1	ARG43	L1
2	ASN46	L2	ASN46	L1	-	-
3	ARG53	H1	-	-	-	-
4	ASN68	H2	ASN68	H2	ASN68	H2
5	SER69	H2	-	-	SER69	H2
6	SER72	H2	-	-	-	-
7	LYS73	H2	LYS73	H2	-	-
8	TYR110	L3	-	-	TYR110	L3
9	ARG111	L3	-	-	-	-
10	ARG113	L3	ARG113	L3	-	-
11	ARG114	L3	ARG114	L3	ARG114	L3
12	LYS115	L3	LYS115	L3	-	-
13	-	-	ALA47	H1	-	-
14	-	-	ARG56	H1	ARG56	H1
15	-	-	-	-	PHE48	H1
16	-	-	-	-	MET49	H1

The table highlights the differential binding of HMG box of SOX2 with A-allele containing DNA and T-allele containing DNA in terms of amino acid residues and SSEs involved in docked complex. Experimentally determined amino acid residues and SSEs are also given for the reference purpose (Remenyi et.al. 2003)

*SSEs* Secondary structure elements, *L* Loop, *H* Helix

Hydrogen bonds (HBs) determine the strength of intermolecular interactions [22]. Therefore, docked complexes of SOX2 ^(HMG)^ with ancestral and derived alleles carrying DNA target sites were analyzed for HBs pattern [23]. In total twelve HBs were noticed for SOX2 ^(HMG)^ and derived A-allele carrying DNA complex (Table 3), whereas ancestral T-allele DNA-SOX2 ^(HMG)^ complex involves only 7 HBs (Table 3). ARG56 and ASN68 of SOX2 ^(HMG)^ formed HBs with C10 and G41 of the ancestral T-allele (Fig. 2B), while ARG43 of SOX2 ^(HMG)^ made contacts with A15 and T37 of derived A-allele (Fig. 3B). Thus, our docking results showed that SOX2 ^(HMG)^ forms a reliable and energetically more favored contact with the derived A-allele containing DNA than it does with the ancestral T-allele containing DNA.

**Table 3:** Hydrogen bond interactions between DNA and HMG box of SOX2 protein determined through molecular docking experiments

Derived A-allele DNA					Ancestral T-allele DNA			
**S.No**	**Interacting residues of SOX2**	**D…A Distance Ȧ**	**D-H…A Distance Ȧ**	**Interacting nucleotides**	**Interacting residues of SOX2**	**D…A Distance Ȧ**	**D-H…A Distance Ȧ**	**Interacting nucleotides**
1	ARG43NH1	3.02	2.64	DT37O2	ARG43NH1	3.68	2.02	DT39O2
2	ARG43NH1	2.95	2.09	DG38O4	ARG43NH1	3.5	2.18	DT39O4
3	ARG43NH2	3.07	1.94	DT16O2	ARG43NH2	2.97	2.14	DA14O3
4	ARG43NH2	2.95	2.32	DT16O4	ARG56NH1	3.75	2.31	DT42O4
5	ASN46ND2	3.8	2.13	DA15O4	ASN68ND2	3	2.21	DC10O2
6	ALA47N	3.68	2.34	DA14O3	ASN68ND2	3.74	2.31	DC10O4
7	ARG56NH1	2.88	2.07	DT40O3	ASN68OD1	2.43	2.05	DG41H21
8	ASN68ND2	2.97	2.47	DG41O3	-	-	-	-
9	LYS73HZ1	2.15	2.22	DA11O3	-	-	-	-
10	ARG113N	3.01	2.09	DT37O3	-	-	-	-
11	ARG114NH2	3	1.85	DG18O3	-	-	-	-
12	LYS115HZ2	2.26	1.76	DA9O5	-	-	-	-

Amino acid numbering is based on position of HMG box domain (39–115) within SOX2 protein. The sequence of bases in one strand of DNA (chain b) are numbered from 1–25 and in the other strand (chain a) are numbered as 26–50. D (A, C, T, G) denotes Deoxyribonucleotides, D…A, denotes distances between donor atom and acceptor atom, while D-H…A illustrates distance between the hydrogen bonded to donor atom and acceptor atom

### Evaluation of dynamic properties of the protein-DNA complexes

To get a deeper insight into the dynamic behavior and binding differences induced by the Homo sapiens-specific evolutionary substitution (T > A), structural stability and flexibility of DNA–protein complexes and the corresponding binding free energies were measured at 100 ns (ns: nanosecond, that is one billionth of a second) trajectories through molecular dynamics (MD) simulation.

**Fig. 4:**
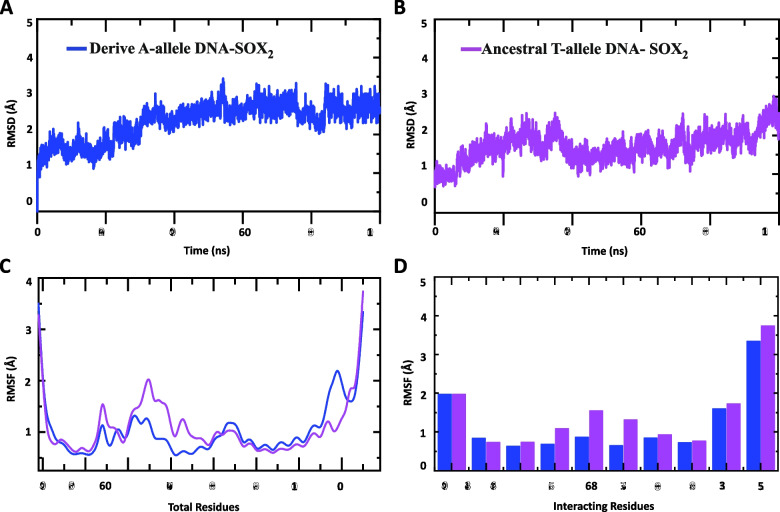
Dynamic stability and residual flexibility of DNA–protein complexes along the course of 100 ns simulation. **A**, **B** The RMSD graphs of both complexes throughout the simulation explaining their stability and equilibration nature. In comparison with ancestral T-allele DNA-SOX2 (violet) complex (**B**), derived A-allele DNA-SOX2 (blue) complex (**A**) appears to be well stabilized. The x-axis shows time in nanoseconds while y-axis show RMSD in Å. **C** RMSF plots for each trajectory file. The x-axis shows total number of residues while y-axis show RMSF in Å. **D** RMSF plots for interacting residues of both complexes. The x-axis shows the interacting residues while y-axis show RMSF in Å. Violet color depict ancestral T-allele DNA-SOX2 complex whereas blue color depict derived A-allele DNA-SOX2 complex

MD simulation-based results suggest that both the complexes, i.e. derived A-allele containing DNA (Homo sapiens)-SOX2 complex and the ancestral T-allele containing DNA (Neanderthal/Denisovans)-SOX2 complex exhibit almost similar average root-mean-square deviations (RMSD) (Fig. 4A and B). However, compared to the ancestral T-allele containing DNA (Neanderthal/Denisovans)-SOX2 complex, derived A-allele containing DNA (Homo sapiens)-SOX2 complex possesses more dynamic stability (Fig. 4A). For instance, the average RMSD for the derived A-allele containing complex was 1.8 Å and the structure did not deviate significantly over the simulation time, but a minor level of conformational deviations from the original static complex structure were observed between 70–80 ns (Fig. 4A). The average RMSD for the ancestral T-allele containing DNA (Neanderthal/Denisovans)-SOX2 complex was comparable with the derived A-allele containing DNA (Homo sapiens)-SOX2 complex (Fig. 4B). However, over the simulation time of 0.00 ns to 100.00 ns the ancestral T-allele containing DNA (Neanderthal/Denisovans)-SOX2 complex revealed abrupt conformational fluctuations in RMSD values which is suggestive of extreme structural perturbation and relatively weaker and unstable intermolecular interactions (Fig. 4B). These data suggest that Homo sapiens-specific single nucleotide substitution within brain exclusive human accelerated enhancer (BE-HAE) hs1210 might have evolved conformationally more stable and efficient interaction between SOX2 ^(HMG)^ and target DNA binding site.

To obtain information on local flexibility and thermal stability of the protein, root mean-square fluctuations (RMSF) are often calculated from molecular dynamics simulations [24]. In context of the macromolecular interactions, the higher RMSF value indicates a more flexible and thus unstable interactions [25]. In contrast, the smaller RMSF values correspond to minimal atomic movements about their average positions during the simulation and hence depict the stable macromolecular interactions [26]. RMSF plot in Fig. 4C exhibits almost a similar trend of residue fluctuation profile for both ancestral and derived alleles based DNA–protein complexes with an average RMSF of 2.5 Å. However, careful analysis revealed that the amino acid residues 65–95 of SOX2 ^(HMG)^ are more stable in complex with Homo sapiens-DNA target site (carrying derived A-allele) when compared to SOX2 complex with DNA target site carrying ancestral T-allele (Fig. 4C). Here, we also measured the RMSF with respect to Cα atom of each interacting residue of HMG box and a plot of RMSF was employed to depict the fluctuations for both ancestral and derived alleles carrying protein-DNA complexes. Figure 4D shows that each interacting amino acid residue of HMG box in derived A-allele containing complex is more stabilized.

**Fig. 5:**
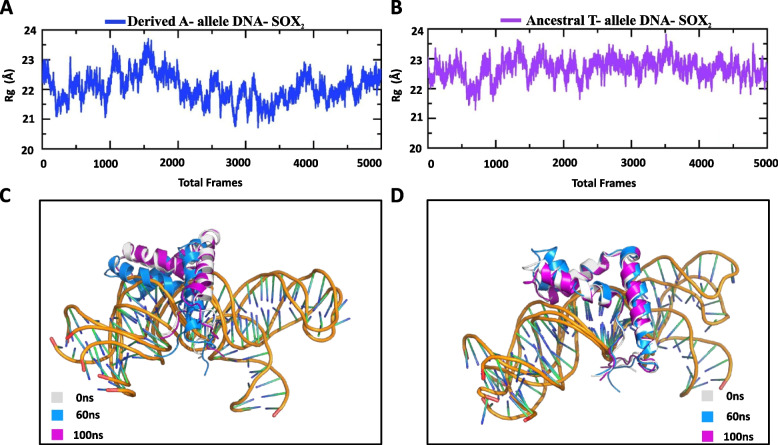
Radius of gyration (RoG) analysis. **A**, **B** RoG plot calculated for derived A-allele DNA-SOX2 (blue) and ancestral T-allele DNA-SOX2 (violet) complexes during the 100 ns simulation. The x-axis shows total number of frames while y-axis shows *Rg* in Å. **C**, **D** Structural superposition of derived A-allele DNA-SOX2 and ancestral T-allele DNA-SOX2 PDBs at different time points. Grey, blue and magenta colors represent 0 ns, 60 ns and 100 ns

A compact packing of amino acid residues is known to affect the stability of macromolecular assemblies [27]. Therefore, we have used the MD simulations to calculate the Radius of gyration (*Rg*) as function of simulation time, which is a measure to estimate the protein structure compactness [28]. Ancestral T-allele containing DNA (Neanderthal/Denisovans)-SOX2 and derived A-allele containing DNA (Homo sapiens)-SOX2 complexes possess substantial differences in the pattern of *Rg* (Fig. 5A and B). For instance, the average *Rg* value for derived A-allele based complex was 22.0 Å, whereas the average *Rg* value for ancestral T-allele based complex was observed to be 23.0 Å. The lower *Rg* value suggests the tightest and most stable packing of derived A-allele based DNA-SOX2 complex compared to ancestral T-allele based DNA–protein complex (Fig. 5A and B).

Temporal aspects of structural stability of protein-DNA complex were investigated by alignment of PDB structures at different time points. These data revealed that at simulation time of 60 ns the HMG box physically moved inside the major grove and thereby favored tightly packed binding with derived A-allele containing DNA target site, whereas at the simulation time of 0 ns and 100 ns the loose interaction was observed between HMG box and derived DNA (Fig. 5C). However, at all set time points (0 ns, 60 ns and 100 ns), HMG box failed to tightly intercalate into the major groove of ancestral T-allele containing DNA (Fig. 5D).

**Fig. 6:**
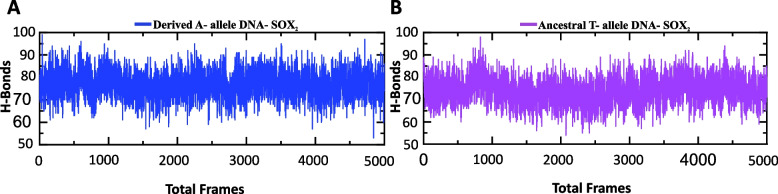
Molecular dynamics simulation based Inter- and Intramolecular hydrogen bonds analysis. **A**, **B** Hydrogen bond plots for derived A-allele DNA-SOX2 (blue) and ancestral T-allele DNA-SOX2 (violet) complexes during the 100 ns simulation. The x-axis shows total number of frames while y-axis shows H-bonds count

To provide further insights into the binding affinities of HMG box for ancestral and derived alleles containing target sites, hydrogen bonding (HB) differences between the two complexes were evaluated by using 5000 structural frames obtained from MD simulation during the 0.00 ns to 100.00 ns. During the simulation time, significant rearrangements in intermolecular HBs were observed between the two complexes (Table 4). In case of derived A-allele containing DNA, 12 hydrogen bonds were observed between HMG box and target DNA site before the MD simulation (0.00 ns) whereas during the MD simulation the HB network readjustments were seen with formation of three extra bonds with the DNA molecule through Arg40, Lys42 and Arg98 residues of HMG box (Table 4). It can be seen that in terms of intermolecular HB networks Arginine residues of HMG box are major contributors in making links with derived A-allele containing DNA (Table 4). Only 7 intermolecular HBs were observed between HMG box and ancestral T-allele containing DNA (Neanderthal/Denisovans) before the MD simulation while during the MD simulation hydrogen bonds number was increased to 12, with extra contributions from Arg 98 and Arg113 residues of the HMG box (Table 4). These differences in intermolecular HB patterns clearly shows that HMG box of SOX2 interacts more robustly with derived A-allele containing DNA complex than to ancestral T-allele containing DNA (Table 4). To further evaluate the overall strength of protein-DNA interaction, the total number of HBs (both inter- and intramolecular hydrogen bonds) within each complex were evaluated during the 0.00 ns to 100.00 ns simulation (Fig. 6A and B). In total 85 HBs were detected for derived A-allele containing DNA-HMG complex, whereas 75 hydrogen bonds were detected for ancestral T-allele containing DNA-HMG complex (Fig. 6A and B). These results further validate the enhanced interaction of HMG box of SOX2 with Homo sapiens-specific substitution carrying target DNA site through conformational changes in protein-DNA complex.

**Table 4:** Hydrogen bonds between the SOX2 protein and DNA before and after MD simulation

**Complex name**	**Before simulation**				**After simulation**		
	**Index**	**SOX2**	**Dist. [Å]**	**Human DNA**	**SOX2**	**Dist. [Å]**	**Human DNA**
**Derived A-allele** **DNA-SOX2**	1	ARG43	3.02	DT	ARG40	1.81	DG
	2	ARG43	2.95	DG	LYS42	1.81	DT
	3	ARG43	2.95	DT	ARG43	1.93	DT
	4	ARG43	3.07	DT	ARG43	1.76	DT
	5	ASN46	3.8	DA	ARG43	2.03	DG
	6	ALA47	3.68	DA	ASN46	2.98	DA
	7	ARG56	2.88	DT	ARG56	1.73	DG
	8	ASN68	2.97	DG	ASN68	2.2	DT
	9	LYS73	2.15	DA	LYS73	1.83	DC
	10	ARG113	3.01	DT	LYS80	1.75	DC
	11	ARG114	3	DG	ARG98	2.11	DT
	12	LYS115	2.26	DA	ARG98	1.69	DT
	13	-	-	-	ARG113	2.28	DT
	14	-	-	-	ARG113	2.32	DG
	15	-	-	-	ARG113	1.76	DT
	**Before simulation**				**After simulation**		
**Ancestral T-allele** **DNA -SOX2**	**Index**	**SOX2**	**Dist. [Å]**	**Neanderthal DNA**	**SOX2**	**Dist. [Å]**	**Neanderthal DNA**
	1	ARG43	3.5	DT	ARG40	1.93	DT
	2	ARG43	3.68	DT	LYS42	1.9	DG
	3	ARG43	2.97	DA	ARG43	2.06	DT
	4	ARG56	3.75	DT	ASN68	1.73	DT
	5	ASN68	3.74	DC	LYS73	2.3	DC
	6	ASN68	3	DC	LYS73	1.93	DT
	7	ASN68	2.43	DG	LYS87	1.83	DC
	8	-	-	-	ARG98	1.75	DA
	9	-	-	-	ARG98	1.63	DA
	10	-	-	-	ARG113	2.26	DG
	11	-	-	-	ARG113	2.09	DT
	12	-	-	-	ARG113	1.82	DT

The table depict inter-molecular hydrogen bonding differences between the two complexes based on 5000 structural frames obtained from molecular dynamics (MD) simulation during the 0.00 ns to 100.00 ns. Dist. Å (angstrom) illustrates distance between the hydrogen bonded donor atom and acceptor atom or length of hydrogen bond. D (A, C, T, G) denotes Deoxyribonucleotides

For each complex (ancestral and derived) binding free energy was evaluated by using 5000 structural frames obtained from MD simulation. Detailed comparison of the energetic profiles of two complexes is given in Table 5. Taken together, the total free energy of binding for the derived A-allele containing DNA (Homo sapiens)-SOX2 ^(HMG)^ complex is more favorable (-140.20 kcal/mol) than the total free energy for the ancestral T-allele containing DNA (Neanderthal/Denisovans)-SOX2 complex (-100.38 kcal/mol) (Table 5). These energetic profiles based on Molecular dynamics simulation analysis are consistent with the protein-DNA docking results which also suggest the tighter binding of SOX2 with the Homo sapiens-specific derived A-allele containing DNA (Table 1).

**Table 5:** Molecular Mechanics/Generalized Born Surface Area (MM/GBSA) based free energy calculations

Complex name	vdW	Electrostatics energy	GB	ESURF	Total binding energy
Derived A-allele DNA- SOX2	-143.98	-9817.95	9840.11	-18.36	**-140.20**
Ancestral T-allele DNA-SOX2	-94.68	-9022.54	9030.37	-13.52	**-100.38**

## Discussion

We performed an electrophoretic mobility shift assay (EMSA) to determine if substitutions at the SOX2 binding site within BE-HAEs hs1210 have any effect on protein binding (Fig. 1C). We found that despite of single nucleotide variation among respective binding sites, SOX2 protein binds to both Homo sapiens and archaic hominins (Neanderthal/Denisovan) based DNA probes (Fig. 1C). However, rigid docking and molecular dynamics simulations based computational data suggests that there are significant geometric and energetic differences in the binding of HMG box of SOX2 with its cognate DNA target sites differed by single residue between archaic hominins and Homo sapiens versions of BE-HAEs hs1210 (Fig. 1A). For instance, rigid molecular docking revealed that SOX2 HMG box grips minor groove and makes specific contact with cognate target site (5′-TACAATG-3′) containing ancestral T-allele in Neanderthal/Denisovan DNA (Fig. 2C and Table 2). In contrast, SOX2 HMG box binds to slightly different residues within the target site (5′ACAAT 3′) carrying derived A-allele in Homo sapiens DNA sequence involving minor groove (Fig. 3C and Table 2). Evaluation of DNA–protein interactions based on rigid molecular docking and MD simulation revealed that the derived A-allele carrying DNA-SOX2 complex involved an increased number of hydrogen bonds than the ancestral T-allele carrying DNA-SOX2 complex (Tables 3 and 4). Prediction of protein − DNA binding free energy interactions using MD simulations revealed an energetically favorable interaction between derived A-allele DNA-SOX2 complex than that for the ancestral T-allele carrying DNA-SOX2 complex (Table 5). Taken together, EMSA-based in vitro experiments, molecular docking, molecular dynamics simulations and free energy calculations suggest that the Homo sapiens-specific SNV (T > A) within DNA target site of SOX2 HMG box did not abolish the DNA–Protein interactions but instead significantly enhanced the affinity of SOX2 protein for its target DNA site by altering three-dimensional geometry and energetics of nucleoprotein complexes. These results fit well with the previously published work showing that SOX2 HMG box can alter gene transcription patterns based on its ability to manipulate DNA geometry [20].

Several lines of evidence suggest that the nucleotide substitution (at position Chr 2; 66,535,938, GRCh38/hg38) within SOX2 binding site of functionally confirmed brain exclusive enhancer, which differs between Homo sapiens and Neanderthals/Denisovans, can alter the regulatory activity of BE-HAEs hs1210. First, it is located in the non-coding region that has already been characterized as forebrain exclusive enhancer via transgenic mice assay [19, 29] (Fig. 1B). Second, the DNA region where the substitution occurs is conserved over mammalian evolution and has previously been characterized as human accelerated enhancers (HAEs) that are likely to regulate human-specific traits [18]. Third, it falls at the 6th position within the 15-bp sequence motif identified as a target site for the transcription factor SOX2 (TAGACA^*^ACAATGGAT), known to play a vital role in neural development (Fig. 1A). Fourth, using human population genetic data it has been shown that the derived allele inhabiting Homo sapiens-specific binding motifs of SOX2 is under positive selection in human populations, implicating further the role of this region in evolution of Homo sapiens-specific traits [18]. Fifth, in the present study we showed that the DNA sequence where the substitution occurs binds SOX2 in vitro. Sixth, comparison of the DNA-binding energetic reveals that there is a difference in the mechanism of binding of SOX2 with ancestral and derived alleles, potentially creating transcriptional activity differences between variant alleles in forebrain tissues. Previous studies have shown that single-nucleotide substitutions within the DNA binding site of SOX2 results in a different level of transcription through changes in correct three-dimensional geometry of nucleoprotein complexes [20]. Furthermore, higher affinity of SOX2 with its target binding sites has been associated with long-lived binding that contributes to more pluripotent progeny in developing mouse embryo whereas the weaker DNA-SOX2 interaction is known to decrease long-lived binding, SOX2 target genes expression, and pluripotent cell numbers [30]. These investigations concluded that the SOX2-DNA binding affinity determines the mammalian cell fate as early as the four-cell stage [30]. Therefore, it is conceivable to suggest that evolution of higher affinity of SOX2 for its target binding site within BE-HAEs hs1210 might have resulted in more robust transactivation of respective target genes in the forebrain tissues of Homo sapiens after they diverged from archaic humans (Neanderthals and Denisovans) some 450,000 years ago [31]. Conceivably, such small scale changes in TFBSs of master developmental regulators such as SOX2 might have been instrumental in evolving differences in brain physiology and anatomy between Homo sapiens lineage and archaic hominins (Neanderthals and Denisovans) and between hominins and great apes. However, a fuller understanding of how different binding affinity of SOX2 at the ancestral site (Neanderthals /Denisovans) and at the derived position on the human Chr 2; 66,535,938 (GRCh38/hg38) affects transcription awaits further studies in model systems.

Gains and losses of TFBSs are widespread and are known to have profound effects on organismal development. Exemplars documenting this mode of change include changes in the regulation of Sonic hedgehog (Shh) gene in the loss of limbs in snakes [32], changes in TBX5 regulation in evolution of fish fins [33], changes in PAX3/PAX7 regulation in craniofacial evolution in humans [34], changes in GDF6 regulation in the evolution of the human foot [35] and changes in GADD45G and FZD8 regulation and evolution of mammalian brain size [36, 37]. It is noticeable that evolutionary rewiring of transcription circuitry does not require only gains and losses of TFBSs but can also entails differences in binding affinities of existing TFs with their target sites through changes in three-dimensional geometry of nucleoproteins complexes and binding energetics. In one such example, positions in the human genome sequence that are different from the orthologous positions in archaic hominins (Neanderthals and Denisovans) have been associated with differential TF binding affinity and consequently the evolution of traits unique to Homo sapiens lineage such as modern language [15]. The present study demonstrates that positively selected Homo sapiens-specific nucleotide variant within the non-coding intragenic regulatory HAR hs1210 has increased the DNA binding affinity of SOX2 through changes in the three-dimensional geometry and binding energetics of the nucleoproteins complex. Because the hs1210 expressed the reporter gene exclusively in the forebrain of transgenic mice, more specifically in lateral ganglionic eminence (LGE), a transient structures in the developing telencephalon (Fig. 1B), it is tempting to speculate that the substitution at position Chr 2; 66,535,938 (GRCh38/hg38) in intragenic region of chromosome 2 might have been involved in the evolution of forebrain. It is noteworthy that this derived nucleotide variant in Homo sapiens is not present in Neanderthals and Denisovans (Fig. 1A). Thus, it is possible that this increase in affinity of SOX2 for its target DNA site might have altered the forebrain specific expression in Homo sapiens lineage, after their split from archaic hominins approximately 550,000–750,000 years ago [10, 38]. These findings are in line with previous studies, that demonstrate that the certain sub-anatomical regions of the forebrain have evolved after the split of Neanderthals and Homo sapiens, most prominently parieto-temporal lobe of the neocortex has increased and orbitofrontal cortex is wider in Homo sapiens as compared to Neanderthals [39, 40].

We deployed a combination of in vitro and computational approaches to show precisely how the SOX2 protein binds more efficiently to its putative binding site containing a positively selected nucleotide position derived in Homo sapiens than does the ancestral allele in Neanderthals and Denisovans. Based on these results, one may speculate that these non-coding single nucleotide changes in regulatory regions that are unique to Homo sapiens lineage could potentially be involved in the evolution of gene expression differences between archaic hominins and Homo sapiens. In this case, evolution of Homo sapiens-specific traits might not entail major transformations in regulatory architecture in terms of gain and loss of TFBSs. Instead, major differences in gene expression patterns and consequently the trait differences between archaic hominins and Homo sapiens might have involved the evolution of the affinity differences of TFs for their target DNA sites. Thus, our data offers general insights into how the functional diversification of cis-regulatory regions through changes in TFs binding affinities contributes to evolutionary novelty and the origin of differences between the two sibling species such as archaic hominins and Homo sapiens.

## Conclusions

In this work we investigated the evolutionary significance of non-coding regulatory HAR, i.e. BE-HAE hs1210 [18]. This HAR functions as a forebrain exclusive enhancer and contains positively selected human-specific nucleotide change that has arisen after the split between Homo sapiens and archaic human lineages [19]. Here we used the combinations of in vitro and bioinformatics analysis to comparatively characterize the evolutionary significance of positively selected Homo sapiens-specific substitution (T > A) within BE-HAEs hs1210. Our comparative molecular structural analysis showed that Homo sapiens-specific single nucleotide substitution has increased the affinity of a SOX2 transcriptional factor for its target binding site within BE-HAE hs1210. These findings suggest that this predicted enhanced affinity of SOX2 towards its target site could drive the target gene expression more robustly within forebrain of Homo sapiens compared with the archaic humans or alternatively within novel territories in the forebrain of Homo sapiens. However, further experimental studies will be necessary to confirm whether in fact these changes in transcriptional factor binding affinity translate into functional modifications of gene expression.

## Materials and methods

### SOX2 expression and verification

A gene encoding SOX2 was amplified using gene specific primers (forward primer: 5’ CATGATGGAGACGGAGCTG 3’; reverse primer: 5’ TGTGTGAGAGGGGCAGTGT 3’) and cloned into the pET30a vector as an N-terminal hexa-histidine fusion, using standard molecular biological approaches (Additional file 1). The resultant vector sequence was verified for the presence of any point mutations generated. This recombinant reporter expression construct was transformed into BL21(DE3) E. coli cells for expression. A saturated overnight culture was inoculated into 5 ml of LB medium supplemented with 50 µg/mL kanamycin at a 1:100 dilution and grown at 37 °C with continuous shaking until the OD600 reached 0.6 ~ 0.8. Protein expression was induced at 1 mM isopropyl β-D-1-thiogalactopyranoside (IPTG) at 37 °C for 4 h. The cells were pelleted at 2400 × g, re-suspended in lysis buffer (50 mMTris, pH 7.5, 300 mMNaCl, 0.5 mg/mL lysozyme), and lysed by flash freezing in liquid nitrogen and thawing at 37 °C three times prior to incubation for 1 h with 20 U/mL DNase I. The resulting lysate was pelleted at 22,500 × g and the supernatant discarded. The pelleted inclusion bodies containing SOX2 were washed three times with wash buffer (50 mMTris, pH 7.5, 300 mMNaCl, 0.5% Triton X-100) prior to resuspension in denaturing and loading buffer (4% SDS, 10% 2-mercaptoethanol, 20% glycerol, 0.004% bromophenol blue and 0.125 M TrisHCl). This mixture was heated at 95 °C for 5 min and then load directly to the gel (Additional file 2).

### Electrophoretic mobility shift assay

The following oligonucleotides and their reverse complimentary strands were synthesized, labeled with biotin, annealed, and used as probes for EMSA analysis: oligonucleotides (derived A-allele containing probe: 5′-GCTTAGACAACAATGGATAAAGAG-3′ and 5′-CGAATCTGTTGTTACCTATTTCTC-3′; ancestral T-allele containing probe: 5′- TAGCTTAGACTACAATGGATAAAG -3′ and 5′-ATCGAATCTGATGTTACCTATTTC-3′), carrying the substitutions in enhancer region of human and neanderthal respectively [41]. EMSA was carried out with the Gel Shift Kit (Viagene). In brief, the double-stranded probes (20 fmol) were incubated with purified SOX2 at room temperature for 20 min in the presence of 100 mMTris (pH 7.5), 500 mMKCl, and 10 mM DTT in a 20 mL reaction. A competitive binding test was performed under the same condition with the addition of a 100-fold excess of unlabeled double-stranded oligonucleotides. For the supershift assay, 1.0 mg anti-SOX2 antibody was added and incubated on ice for 30 min. DNA–protein complexes were resolved on 6.0% (wt/vol) native polyacrylamide gels (Bio-Rad), transferred to Biodyne nylon membranes (Pierce), viewed under Ultraviolet transilluminator (UV transilluminator), and photographs of the gel were taken using Dolphin gel documentation system (Wheeltech, USA) (Fig. 1C and Additional file 3).

### Sequence acquisition and comparative analysis

Protein and DNA sequences of human (*Homo sapiens)*, primates *(Pan troglodytes, Gorilla beringei, Pongo abelii, and Galago),* non-primate mammals *(Mus musculus, Rattus norvegicus, Loxodonta Africana, Oryctolagus cuniculus, Equus caballus, Monodelphis domestica, Callithrix jacchus, Lama pacos, Canis lupus familiaris, Felis catus and Ornithorhynchus anatinus)* obtained from Ensembl genome browser and the orthologous sequences of archaic human extracted from Neanderthal Genome Browser (http://neandertal.ensemblgenomes.org/index.html) were subjected to multiple sequence alignment (MSA) through ClustalW [42, 43]. The resultant MSA was analyzed to determine the conserved segments (Fig. 1A and Additional file 4).

### DNA and protein modeling

We generated 3D structural models of DNA from the enhancer sequence carrying the derived A-allele (5’TTAGACA*ACAATGGATA 3’) and ancestral T-allele (5’ TTAGACT*ACAATGGATA 3’) using 3D-DART provided by the High Ambiguity Driven protein–protein DOCKing (HADDOCK) web-server (http://haddock.science.uu.nl/services/3DDART/) followed by energy minimization. It provided a perfect B‐DNA structure of the desired sequence, which was convenient for the structural study of DNA in complex with proteins [44]. The crystal structures of the human HMG domain (amino-acids; 39–121) of SOX2 (PDB ID: 1O4X) were retrieved through Protein data bank PDB [22]. 3D structures were analyzed using the University of California, San Francisco (UCSF) Chimera extensible molecular modeling system package (Version 1.11.2) [45].

### DNA–protein docking and refinement

HADDOCK (version 2.2), an online web server, was used for protein-DNA docking [46, 47]. HADDOCK docks 1000 structures in a rigid body minimization (it0) mode and refines the top 200 in a semi-flexible refinement in the torsion angle space (it1) followed by explicit solvent refinement (water) (the most favorable cluster was listed first) [46]. The HADDOCK approach required near-native complexes for satisfactory results, such as the active and passive sites of the DNA and protein were predicted using the CPORT tool [48]. To study the interactions between amino acids of SOX2, human and Neanderthal DNA directly, the DNA binding domain of SOX2 was docked onto either the A-allele or the T-allele. The initial complex was further refined by the maximum likelihood method in REFMAC5 [49].

### Protein-DNA interaction analysis

DNAproDB is a web-based visualization tool for structural analysis of DNA–protein complexes [50]. Herein, we used DNAproDB to visualize our docked complexes and to understand the interaction pattern. PDBe-PISA server (www.ebi.ac.uk/pdbe/pisa/) was used to analyze interface of the docked complexes under default criteria [23].

### Molecular dynamics simulation

To explore the dynamic binding features for wild type and mutant alleles based complexes all-atoms biomolecular simulation was performed for the solvated systems using FF19SB force-field in AMBER20 [51]. For DNA OL15 force field was used. To neutralize the effect of any charge on the system counter ions were added. Gentle energy minimization protocol was carried out at 12,000 and 6000 steps to correct any unfavorable bond lengths and angles, and to eliminate unacceptable steric clashes, followed by heating of each system at 300 K for 200 ps (ps: picosecond, that is one trillionth of a second). Weak restraint was used for density equilibration for 2 ns, while the whole system was kept at a constant pressure for 2 ns. A 100 ns MD under constant pressure was performed. For the temperature control, Langevin thermostat (1 atm, 300 K) was used [52]. Particle Mesh Ewald (PME) algorithm was used to compute long-range interactions [53, 54]. The cut-off distances for the different bonds were set at 10 Å. Covalent bond parameterization was performed with the SHAKE algorithm [55]. GPU accelerated simulation using PMEMD. CUDA was used for all the processes. Post-MD trajectories were subjected to thermodynamic stability evaluation, residual flexibility, structural compactness and hydrogen bonding analysis using CPPTRAJ and PTRAJ modules of Amber [56].

### Binding free energy estimation

To determine the impact of evolutionary substitution in the system in terms of binding energy, we used MMPBSA.PY script to reveal the binding differences of HMG box to ancestral (Neanderthal/Denisovans) and derived (Homo sapiens) DNA target sites [26]. A 100 ns trajectory having 5000 structures were used to calculate the BFE using the following equation:

$$\triangle G_{bind}\:=\:\triangle G_{complex}\:-\:\lbrack\triangle G_{receptor}\:+\:\triangle G_{ligand}\rbrack$$

Each term in the binding free energy was estimated using the following equation:

$$G\:=\:G_{bond}\:+\:G_{ele}\:+\:G_{vdW}\:+\:G_{pol}\:+\:G_{npol}$$

The aforementioned equation represents the non-polar, electrostatic, polar, solvent-accessible surface area (SASA), and van der Waals interactions (vdW), respectively .

## Supplementary Information

**Additional file 1.** Electropherogram of PCR products of SOX2. 1% agarose gel stained by ethidium bromide shows PCR products of SOX2.M represents Molecular Marker (100 bp) and S represents samples.

**Additional file 2.** Expression of SOX2 protein.

**Additional file 3.** Full-size image of EMSA/Gel shift assay. Electrophoretic mobility shift assay shows shift in the mobility of SOX2 protein-DNA complexes as compared to the free probes. Modern Human (MH) and Archaic Hominin (AH). Ab indicates Antibody. This image is full-size, uncropped version of Figure 1C.

**Additional file 4.** Evolutionary Conservation of SOX2 Protein. Domain organization of SOX2 protein depicting highly conserved Homeobox domain (HMG). Dots indicate amino acid residues identical to human. a1, a2 and a3 show helices of the HMG domain.

## Data Availability

The datasets analyzed during the current study are available in the Ensembl genome browser(http://www.ensembl.org), NCBI database (https://www.ncbi.nlm.nih.gov/), VISTA Enhancer Browser (https://enhancer.lbl.gov/) and Protein data bank (PDB) (http://www.rcsb.org/pdb).

## Abbreviations

HARs: Human Accelerated Regions
SOX2: SRY (sex determining region Y)-box 2
HMG: High Mobility Group
TF: Transcription Factor
TFBSs: Transcription Factor Binding Sites
EMSA: Electromobility Shift Assay
MSA: Multiple Sequence Alignment
3D. DART: 3DNA-Driven DNA Analysis and Rebuilding Tool
PDB: Protein Data Bank
PME: Particle Mesh Ewald
kDa: Kilodalton
LRH: Long Rang Haplotype
CNS: Central Nervous System
BFE: Binding Free Energy
MD: Molecular Dynamics
RMSD: RootMean-Square Deviation
RMSF: Root Mean-Square Fluctuations
BE-HAEs: Brain Exclusive Human Accelerated Enhancers
