# Supplementary material for: Evolutionary relevance of single nucleotide variants within the forebrain exclusive human accelerated enhancer regions
Source: BMC Mol Cell Biol. 2023 Mar 29;24:13. doi: 10.1186/s12860-023-00474-5 (PMC10053400; doi:10.1186/s12860-023-00474-5)
Supplement: Supplementary file 1 — Additional file 1. Electropherogram of PCR products of SOX2. 1% agarose gel stained by ethidium bromide shows PCR products of SOX2.M represents Molecular Marker (100 bp) and S represents samples. [file 12860_2023_474_MOESM1_ESM.pdf]

Supplementary Figure S1

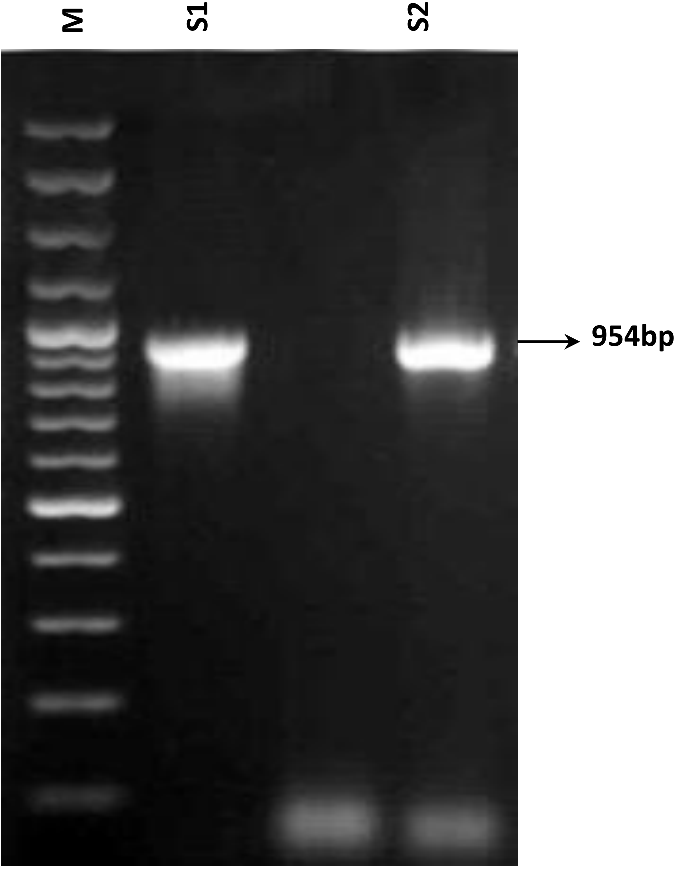

Electropherogram of PCR products of SOX2

1% agarose gel stained by ethidium bromide shows PCR products of SOX2. M represents Molecular Marker (100 bp) and S represents samples
