# Supplementary material for: Evolutionary relevance of single nucleotide variants within the forebrain exclusive human accelerated enhancer regions
Source: BMC Mol Cell Biol. 2023 Mar 29;24:13. doi: 10.1186/s12860-023-00474-5 (PMC10053400; doi:10.1186/s12860-023-00474-5)
Supplement: Supplementary file 2 — Additional file 2. Expression of SOX2 protein. [file 12860_2023_474_MOESM2_ESM.pdf]

Supplementary Figure S2

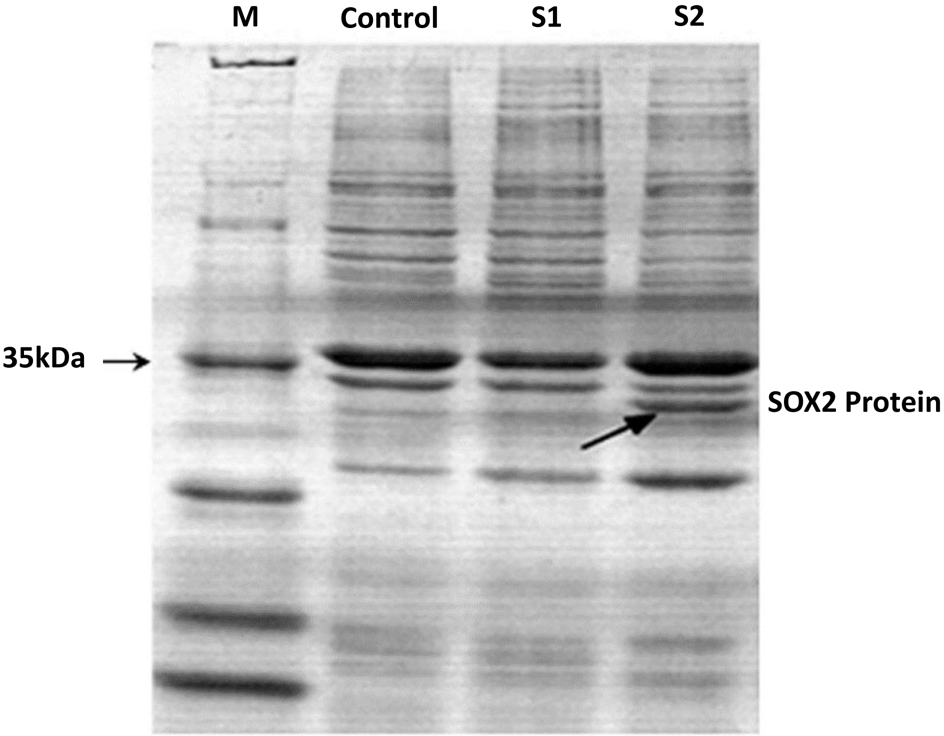

Expression of SOX2 protein SDS-PAGE of SOX2 protein. M: protein marker 10-245 KDa; control: Bl21 cells; S: samples
