# Supplementary material for: Evolutionary relevance of single nucleotide variants within the forebrain exclusive human accelerated enhancer regions
Source: BMC Mol Cell Biol. 2023 Mar 29;24:13. doi: 10.1186/s12860-023-00474-5 (PMC10053400; doi:10.1186/s12860-023-00474-5)
Supplement: Supplementary file 3 — Additional file 3. Full-size image of EMSA/Gel shift assay. Electrophoretic mobility shift assay shows shift in the mobility of SOX2 protein-DNA complexes as compared to the free probes. Modern Human (MH) and Archaic Hominin (AH). Ab indicates Antibody. This image is full-size, uncropped version of Figure 1C. [file 12860_2023_474_MOESM3_ESM.pdf]

Supplementary Figure S3

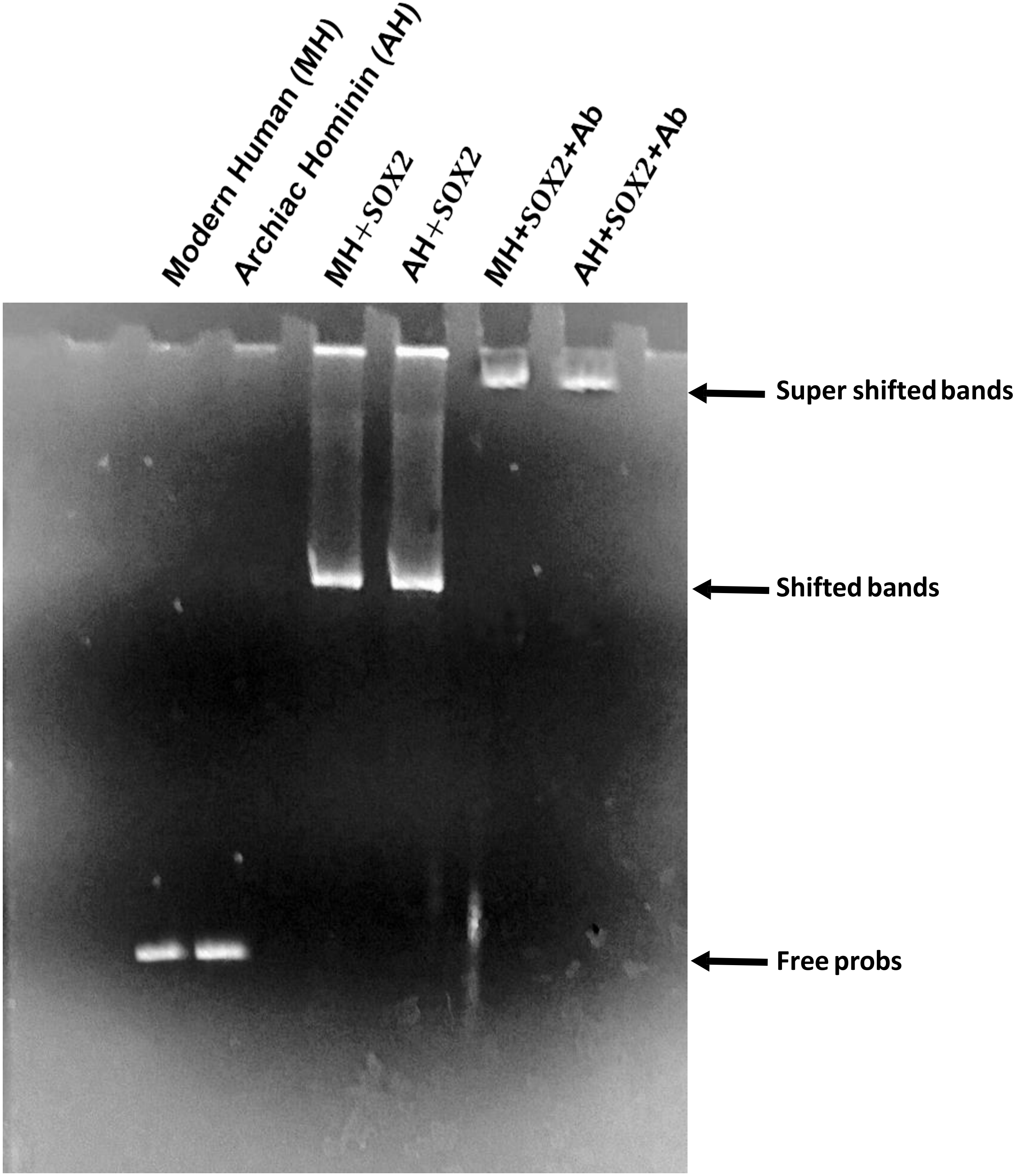

Full-size image of EMSA/Gel shift assay

Electrophoretic mobility shift assay shows shift in the mobility of SOX2 protein-DNA complexes as compared to the free probes Modern Human (MH) and Archaic Hominin (AH). Ab indicates Antibody. This image is full-size, uncropped version of Figure 1C.
