# Supplementary material for: Evolutionary relevance of single nucleotide variants within the forebrain exclusive human accelerated enhancer regions
Source: BMC Mol Cell Biol. 2023 Mar 29;24:13. doi: 10.1186/s12860-023-00474-5 (PMC10053400; doi:10.1186/s12860-023-00474-5)
Supplement: Supplementary file 4 — Additional file 4. Evolutionary Conservation of SOX2 Protein. Domain organization of SOX2 protein depicting highly conserved Homeobox domain (HMG). Dots indicate amino acid residues identical to human. a1, a2 and a3 show helices of the HMG domain. [file 12860_2023_474_MOESM4_ESM.pdf]

Supplementary Figure S4

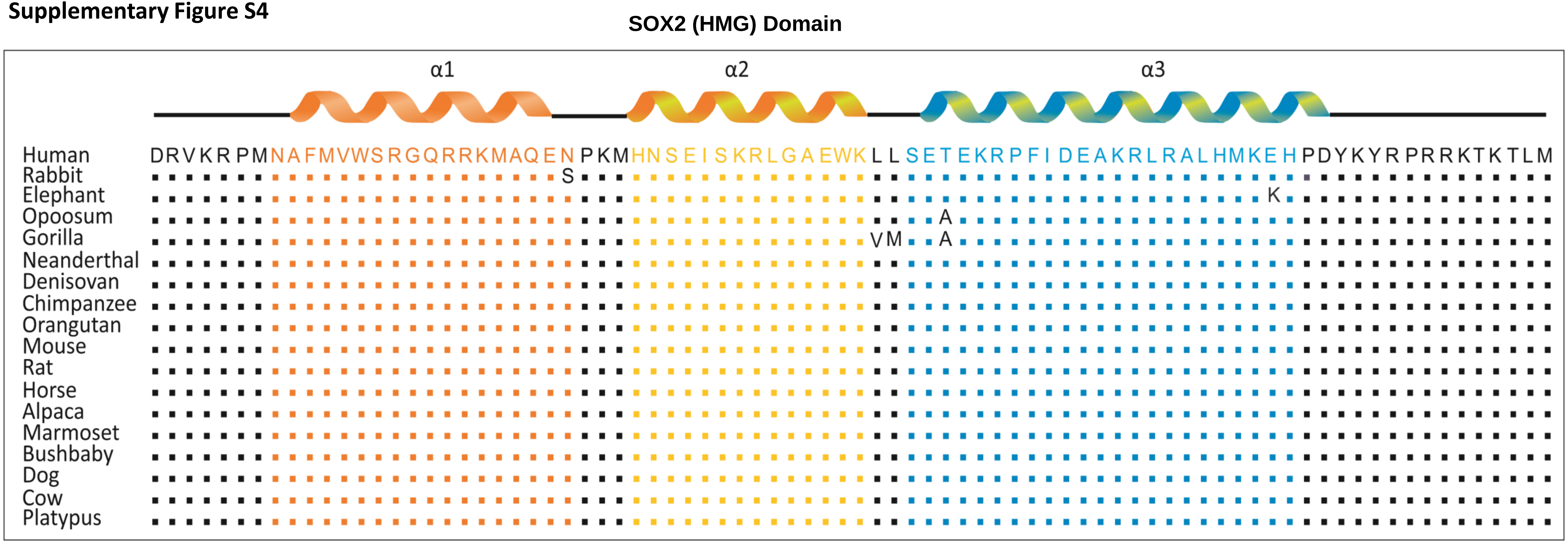

Evolutionary Conservation of SOX2 Protein

Domain organization of SOX2 protein depicting highly conserved Homeobox domain (HMG). Dots indicates amino acid residues identical to Human. a1, a2 and a3 shows helices of the HMG domain.
